# Prognostic value of serum cytokeratin 19 fragments (Cyfra 21-1) in patients with non-small cell lung cancer

**DOI:** 10.1038/srep09444

**Published:** 2015-04-22

**Authors:** Youtao Xu, Lei Xu, Mantang Qiu, Jie Wang, Qing Zhou, Lin Xu, Jian Wang, Rong Yin

**Affiliations:** 1 Department of Thoracic Surgery, Nanjing Medical University Affiliated Cancer Hospital, Jiangsu Key Laboratory of Molecular and Translational Cancer Research, Cancer Institute of Jiangsu Province, Baiziting 42, Nanjing, P.R. China, 210009; 2 The First Clinical College of Nanjing Medical University, Nanjing, 210000, China; 3 Department of Thoracic Surgery, The Affiliated Jiangning Hospital of Nanjing Medical University, No. 168 Dongshan Street Gushan Road, Nanjing 211100, China; 4 The Fourth Clinical College of Nanjing Medical University, Nanjing 210000, China; 5 Department of Oncology, Nanjing Medical University Affiliated Cancer Hospital, Baiziting 42, Nanjing, P.R. China, 210009

## Abstract

The role of serum CYFRA 21-1 level in patients with non-small cell lung cancer (NSCLC) remains to be defined. To re-evaluate the impact of serum CYFRA 21-1 in NSCLC survival, we performed this meta-analysis. Databases were searched to identify relevant studies reported after the publication of a meta-analysis in 2004. Totally, 31 studies with 6394 patients were included in this meta-analysis. The pooled Hazard ratios (HRs) indicated that high CYFRA 21-1 level was associated with poor prognosis on overall survival (OS) in patients with NSCLC (HR = 1.60; 95%CI = 1.36–1.89; *P* < 0.001). The pooled HRs were 2.18 (95%CI = 1.70, 2.80; *P* = 0.347) for patients at stage I–IIIA and 1.47 (95%CI = 1.02, 2.11; *P* < 0.001) for stage IIIB–IV. When stratified by surgical intervention, pooled HRs were 1.94 (95%CI = 1.42–2.67; *P* < 0.001) for studies with surgery and 1.24 (95%CI = 0.79–1.95; *P* < 0.001) for studies without surgery. Significant associations were also found in the patients treated with EGFR-TKIs (HR = 1.83; 95%CI = 1.31–2.58; *P* *= * 0.011) and platinum-based regimen (HR = 1.53; 95%CI = 1.18–1.99; *P* = 0.001). Meta-analysis of CYFRA 21-1 related to PFS was performed and pooled HR was 1.41 (95%CI = 1.19–1.69; *P* < 0.001). Our results indicate that high level of serum CYFRA 21-1 is a negative prognostic indicator of patients with NSCLC.

Lung cancer remains the most frequent cause of cancer death globally. Non-small cell lung cancer (NSCLC) accounts for 80–85% of lung cancers^1^. Surgery is the most promising treatment modality for potential cure. However, even patients with stage I NSCLC suffer a 30% risk of relapse after resection^2^. In addition, a great majority of patients, approximately 80%, are diagnosed in advanced stages^3^. In spite of the improvements in diagnostic and therapeutic techniques on lung cancer over the past few decades, the prognosis is still poor, with an estimated survival of only 15% at 5 years^4^. In recent years, many independent clinical and biological prognostic factors for lung cancer have been reported, such as stage, performance status (PS), age, K-ras oncogene mutations, Ki-67 expression, p16 promoter hypermethylation and excision repair cross-complementing 1 (ERCC1) polymorphism^5^,^6^,^7^,^8^. Correct identification of molecular prognostic factors may contribute to a better understanding of cancer development, clinical outcome, and eventually facilitate the rational selection of therapeutic strategies.

CYFRA 21–1 is a fragment of cytokeratin (CK) 19 and CKs are the principal structural elements of the cytoskeleton of epithelial cells, including bronchial epithelial cells. CK19 is expressed in the unstratified or pseudostratified epithelium lining the bronchial tree, and been reported to be overexpressed in many lung cancer tissue specimens. The CYFRA 21–1 expression patterns in tissues are well-maintained even during the process of transformation of the tissue from normal to tumor tissue^9^,^10^. Many studies demonstrated high expression and diagnostic value of CYFRA 21–1 in NSCLC^11^. Also, some researches reported that CYFRA 21–1 expression was associated with metastatic lymph nodes, TNM stage, tumor size, and differentiation^12^. CYFRA 21-1 has been identified to be a sensitive biomarker in NSCLC. It was reported that the sensitivity of CYFRA 21-1 in diagnosis of squamous cell carcinoma was 62%^13^. Following the fist identification of high serum CYFRA 21-1 level as a valuable prognostic marker in lung cancer patients in 1993^14^, numerous studies have been performed to validate the result. A meta-analysis of the prognostic role of serum CYFRA 21-1 for NSCLC was reported in 2004^15^. They analyzed the data from 1993 to 2001, and demonstrated that a high serum CYFRA 21-1 level was correlated with poor prognosis for NSCLC patients whatever the planned treatment. However, the result of the patients having undergone surgery only showed a trend of statistical significance. Furthermore, this meta-analysis had the following limitations: relatively short follow-ups, including a small number of accrued patients, and not providing detailed treatments for patients who did not undergo surgery.

Subsequently, many studies investigating the role of serum CYFRA 21-1 with more updated therapeutic strategies for NSCLC and larger numbers of accrued patients have been performed^16^,^17^,^18^,^19^,^20^,^21^,^22^,^23^,^24^,^25^,^26^,^27^,^28^,^29^,^30^,^31^,^32^,^33^,^34^,^35^,^36^,^37^,^38^,^39^,^40^,^41^,^42^,^43^,^44^,^45^,^46^. However, these studies yielded conflicting results. Therefore, we conducted an updated meta-analysis using data from these studies to reappraise the effect of serum CYFRA 21-1 on the prognosis in patients with NSCLC.

## Results

### Characteristics of eligible studies

**Figure 1:**
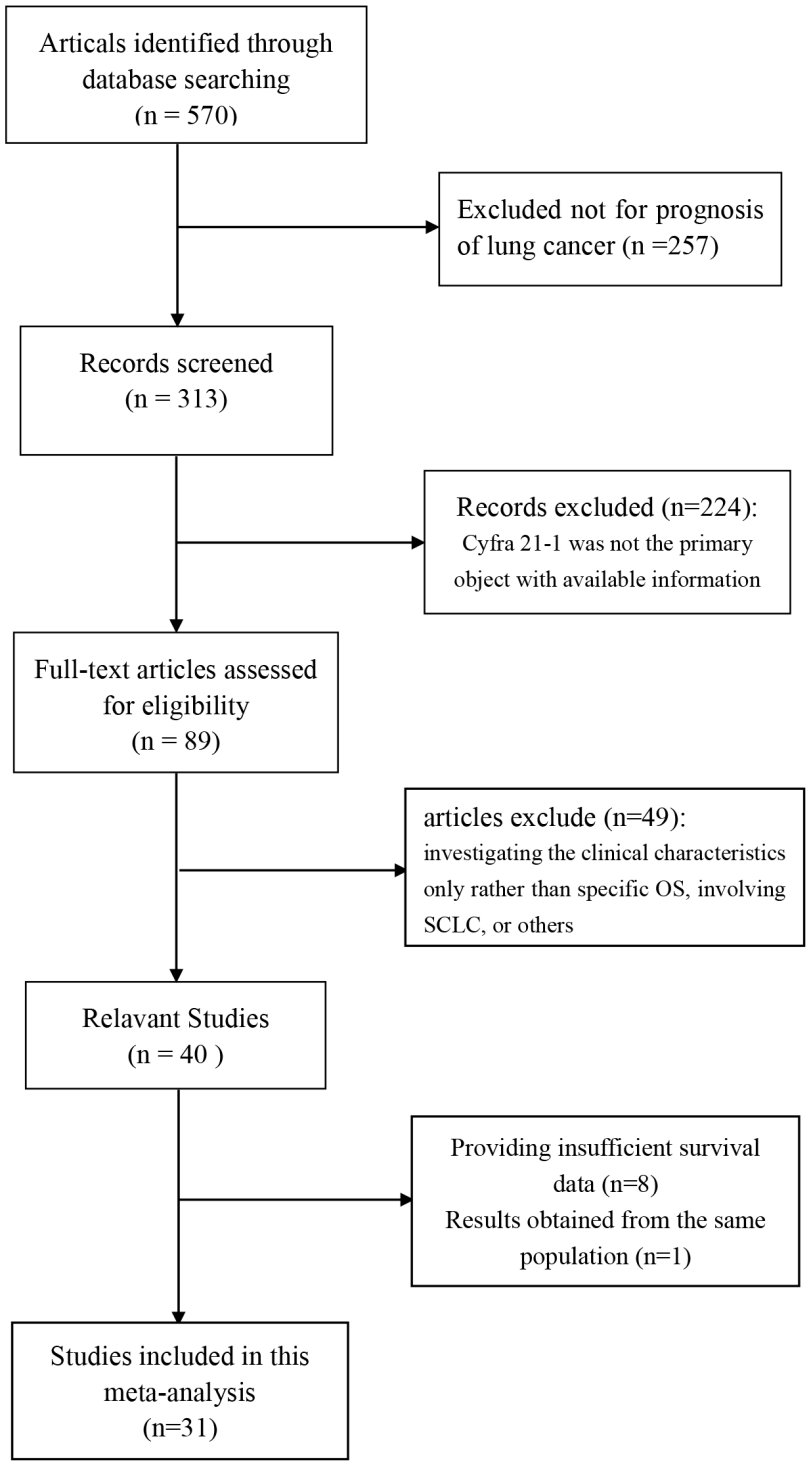
Flow chart of studies in the analysis.

After deleting the duplications, a total of 570 potentially relevant publications were collected after initial search. Among these, 313 articles related to the prognosis of lung cancer were reserved. Then 89 studies primarily researching the CYFRA 21-1 level were selected in full text for further screening after reviewing the abstracts. 49 studies were excluded for only investigating the clinical characteristics rather than specific OS, involving small cell lung cancer (SCLC), or other out of the scope according to the inclusion and exclusion criteria, consequently leaving 40 available for further review. After carefully reading, 31 studies, with a total number of 6394 patients were identified in this meta-analysis (Figure 1). Some studies reported two endpoints, and they were analyzed separately. The main characteristics of the evaluable studies were listed in Table 1.

In the present meta-analysis, statistical calculations for OS were performed in 31 studies. Sample size of studies ranged from 48 to 1202. 17 studies were performed in Asian, 13 studies were in Caucasian, and one study was in mixed populations. 6 studied NSCLC patients at stage I–IIIA, 9 studied patients at stage IIIB–I, and 15 studied the wide range of stage I–IV. 12 were conducted prospectively, 17 were performed retrospectively, and 2 were not available. The numbers of studies reported all of the patients with surgery and without surgery were 9 and 9, respectively. 7 studies including 1061 patients were involved in PFS calculation. The detailed information of them was described in Table 1.

Serum CYFRA 21-1 was dichotomized into high and low levels, and different cut-off value was selected in each study. Most of the studies utilized the manufacturer's instructions, some applied the median or mean levels as cut-off values, and the remaining studied defined the value by themselves or a ROC curve.

### Main results

**Figure 2:**
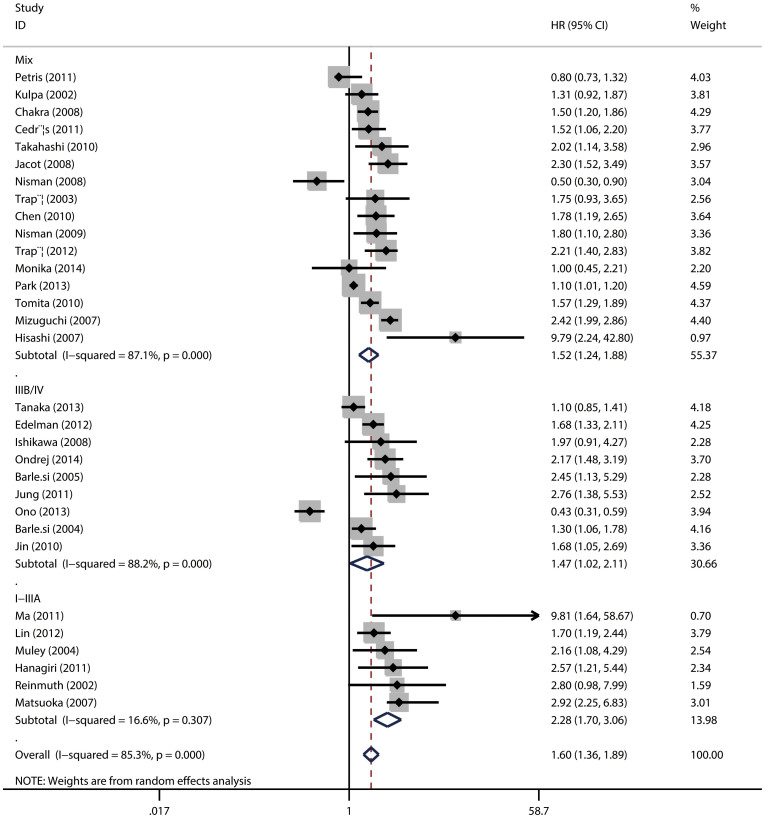
The association between serum CYFRA 21-1 and overall survival of NSCLC stratified by TNM stage.

**Figure 3:**
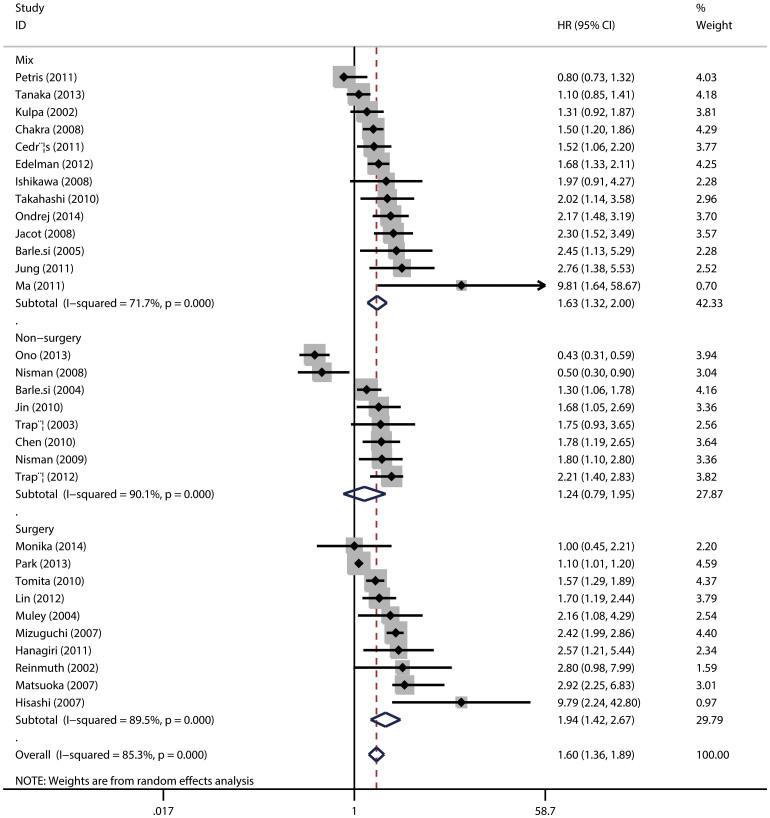
The association between serum CYFRA 21-1 and overall survival of NSCLC stratified by surgical intervention.

The main results of the meta-analysis were listed in Table 2. Among the 31 trials eligible for assessing the relationship between CYFRA 21-1 and OS, the pooled HR was 1.60 (95%CI = 1.36–1.89; *P* < 0.001) (Figure 2), indicating that high serum CYFRA 21-1 level predicted poor OS for NSCLC. In the subgroup analysis by TNM stage, for patients with NSCLC at stage I–IIIA (resected tumor), the pooled HRs were 2.18 (95%CI = 1.70, 2.80; *P* = 0.347) and for stage IIIB–IV (unresectable disease), the pooled HRs were 1.47 (95%CI = 1.02, 2.11; *P* < 0.001) while for mixed stage I–IV, HRs were 1.52 (95%CI = 1.24, 1.88; *P* < 0.001), indicating a significant association between high serum lever of CYFRA 21-1 and poor clinical outcome (Figure 2). Then to evaluate the prognostic roles in surgical intervention, we divided the studies into surgery group and non-surgery group and found a pool HR of 1.94 (95%CI = 1.42, 2.67; *P* < 0.001) for surgery group and 1.24 (95%CI = 0.79–1.95; *P* < 0.001) for non-surgery group (Figure 3).

**Figure 4:**
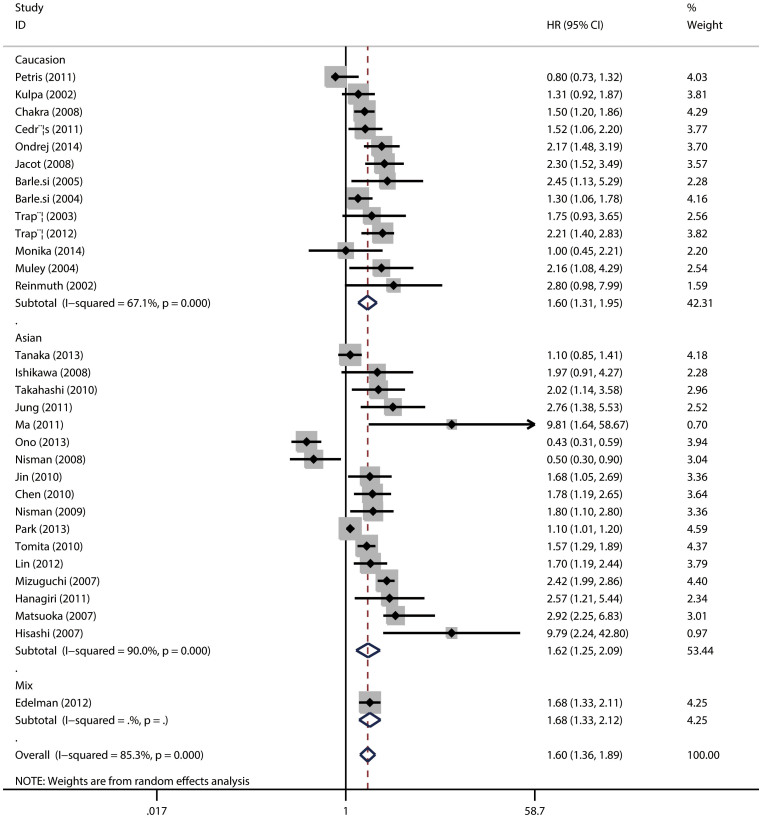
The association between serum CYFRA 21-1 and overall survival of NSCLC stratified by ethnicity.

When stratified by ethnicity, a pooled HR was 1.62 (95%CI = 1.25–2.09; *P* < 0.001) for Asians and 1.60 (95%CI = 1.31–1.95; *P* < 0.001) for Caucasians (Figure 4), favoring the association between high serum CYFRA 21-1 level and poor prognosis. In the subgroup analysis according to study design, a significant link was also found in prospective studies 1.58 (95% CI = 1.27–1.96, *P* *= * 0.001) and retrospective studies 1.75 (95% CI = 1.38–2.22, *P* < 0.001), respectively (Supplementary Fig. S1).

**Figure 5:**
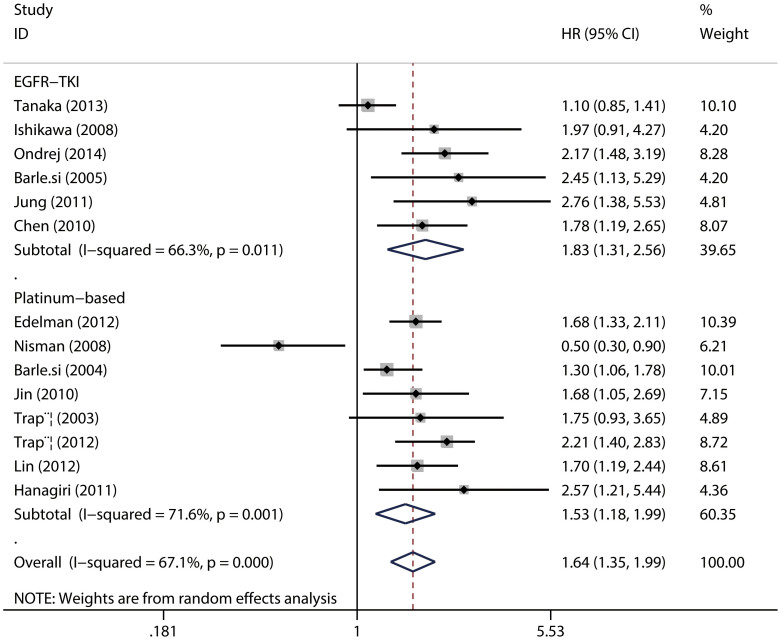
The association between serum CYFRA 21-1 and overall survival of NSCLC stratified by chemotherapy regimen.

When sub-grouped by the chemotherapeutic regimens, the pooled HRs were 1.83 (95%CI = 1.31–2.58; *P* = 0.011) for studies using EGFR-TKIs, and 1.53 (95%CI = 1.53–1.99; *P* = 0.001) for studies applying platinum-based chemotherapy (Figure 5), again, indicating a relationship between high serum CYFRA 21-1 level and poor outcome for NSCLC.

Meta-analysis of PFS was conducted in 7 studies. The pooled HR was 1.41 (95%CI = 1.19–1.69; *P* < 0.001). Statistically significant effect on PFS was found for serum CYFRA 21-1.

We also performed sensitivity analyses by repeatedly deleting the single studies each time from pooled analysis to examine the stability and reliability of meta-analysis results. In our analysis, the omission of individual studies did not materially alter the results because the recalculated ORs and 95%CIs were not quantitatively changed, suggesting that the results were robust and convincing (Supplementary Fig. S2, S3).

### Publication bias

**Figure 6:**
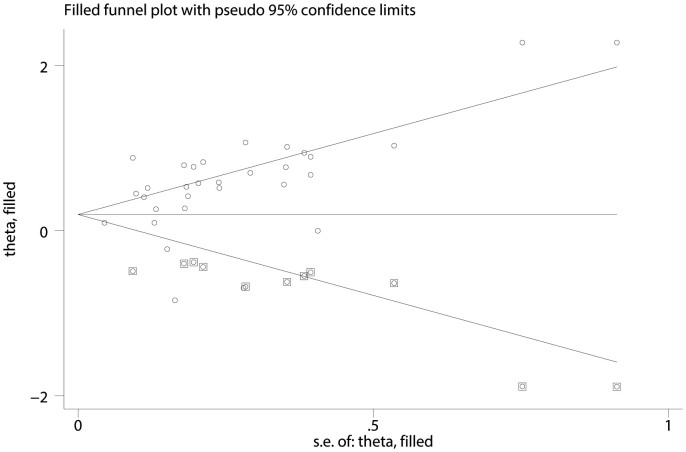
Funnel plot for OS, adjusted with trim and fill method Circles stand for included studies; diamonds stand for presumed missing studies.

Publication bias was assessed by Begg's funnel plot and Egger's test. Publication bias was detected (*P* = 0.144 for Begg's test but *P* *= * 0.03 for Egger's test) for pooled OS. Thus, a trim and fill method was utilized and pooled ORs were recalculated with hypothetically non-published studies to evaluate the asymmetry in the funnel plot^47^ (Figure 6). The recalculated ORs for OS did not change significantly (HR = 1.27; 95% CI = 1.075–1.493; *P* < 0.001), indicating the stability of the results. However, significant publication bias was not discovered among the studies regarding PFS (*P* = 0.368 for Begg's test).

## Discussion

To the best of our knowledge, we demonstrated, for the first time, that high serum CYFRA 21-1 level was an indicator of poor prognosis for NSCLC using updated data. Notably, our meta-analysis included three times more patients than the previously reported one^15^, and the studies employed more updated therapeutic regimen and patients with longer follow-ups. As a result, we were able to show a more plausible result.

In this meta-analysis, we identified 31 eligible studies including 6394 patients and concluded that high serum CYFRA 21-1 level was a poor prognostic indicator for OS and PFS. For different pathological TNM stage, high serum CYFRA 21-1 level was associated with poor clinical outcome of NSCLC patients. Thus, high level of serum CYFRA 21-1 might be a negative prognostic biomarker in NSCLC patients with resected tumor or with unresectable disease. This link was observed in surgery subgroups. Notably, we verified the poor prognostic role of high serum CYFRA 21-1 level in the patients who had undergone surgery (HR = 1.94; 95%CI = 1.42–2.67; *P* < 0.001), while the previous meta-analysis only indicated a trend towards statistical significance (HR = 1.41, 95% CI = 0.99–2.03, *P* = 0.055). However, for patients who did not undergo surgery, the HR was 1.24 (95%CI = 0.79–1.95; *P* < 0.001) while the previous meta-analysis showed high serum CYFRA 21-1 level predicted poor survival (HR = 1.78; 95%CI = 1.54–2.07, *P* < 0.001) in the first year of follow-up^15^.

We also found that high serum CYFRA 21-1 level was a poor prognostic factor for patients treated with EGFR-TKIs or platinum-based regimen. Platinum-based chemotherapy could improve survival and has become the standard chemotherapy for years^48^,^49^, however, the efficacy of platinum-based chemotherapy varies among individuals, with a response rate of 26%–60%^50^. Various tumor markers have been studied in terms of their prognostic or predictive roles in NSCLC patients treated with platinum-based chemotherapy^8^,^51^,^52^,^53^,^54^. However, to date, no serum maker is currently recommended for routine clinical practice in prognosis of NSCLC patients treated with platinum-based chemotherapy. Based on our findings, serum CYFRA 21-1 might be a promising biomarker. EGFR mutations have been reported to be the most important prediction factor for patients treated with EGFR-TKIs^55^. Unfortunately, it is not feasible to obtain an adequate EGFR mutational analysis. Therefore, it is important to identify easily acquired clinical parameters that can serve as surrogate markers for EGFR mutants. Our results suggested that serum CYFRA 21-1 might play such a role in the prediction and prognosis of advanced NSCLC treated with EGFR-TKIs. Notably, this was the firstly pooled analysis to verify the prognostic role of serum CYFRA 21-1 in the NSCLC patients treated with platinum-based chemotherapy or EGFR-TKIs. In consideration of the relatively small sample size, large scale prospective studies are needed to further confirm the results.

The sub-group analyses by ethnicity and study design yielded the same results, further indicating the poor prognosis role of high serum CYFRA 21-1 level in NSCLC.

Attention must be paid to the relatively large heterogeneity among trials. Meta-regression was conducted by ethnicity, surgical intervention, chemotherapy, detective method, cutoff, study design, and sample size. However, none of them was found to be the sources of heterogeneity. In fact, many other factors may also be the potential source of heterogeneity, such as gender, age, and life styles of patients and so on. Due to lack of detailed data, we had to give up performing a meta-regression utilizing these variables. Publication bias is significant threat to the reliability of the results of a meta-analysis. Unfortunately, we did find publication bias by Begg's funnel plot and Egger's test. Then a trim and fill method was adopted to recalculate the adjusted ORs and the results did not change significantly, indicating the stability of the statistic analyses^47^.

Several limitations have to be noted in relation to this meta-analysis. First, all our analyses were based on abstracted data and not on individual patient data (IPD). It was commonly acknowledged that an IPD-based meta-analysis would reproduce more reliable estimation compared with one based on abstracted data^56^, while an IPD-based meta-analysis is a time-consuming effort, especially when some studies without high quality^57^. Second, several HRs were obtained based on the survival curves, which might have biased our results. Third, cutoff values were different among the studies. Finally, the publication bias and heterogeneity in the meta-analysis may partly lessen its statistical power. However, these problems are almost inevitable in a meta-analysis.

In conclusion, our meta-analysis, based on an updated data, found a significant prognostic value of serum CYFRA 21-1. High serum CYFRA 21-1 level is correlated with poor OS and PFS in NSCLC, which might provide a simple and practical method to predict the outcome of NSCLC patients. Interestingly, serum CYFRA 21-1 is also related to the OS in the NSCLC patients treated with EGFR-TKIs or platinum-based regimen. But the sample size is relatively small, and more investigations are needed to identify its prognostic role in this part of NSCLC patients.

## Methods

### Search strategy

To update the data, we intended to exclude the studies accrued in the previous meta-analysis published in 2004^15^. Therefore, only those studies published after January 1, 2002, were eligible. We conducted a computerized literature search of Embase, Web of Science, PubMed and China National Knowledge Infrastructure (CNKI) to identify all the studies that studied the association of serum CYFRA 21-1 lever and lung cancer. The combination of the following key words were used as search terms separately and in combination: “CYFRA 21-1”, “cytokeratin 19”, “non-small cell lung cancer”, “NSCLC”, “prognosis”, “survival”, and “outcome”. Last search was updated on January 08, 2015. The references of all publications and reviews were manually searched to identify potentially relevant studies.

### Inclusion and exclusion criteria

To be eligible for inclusion in this meta-analysis, studies had to meet the following criteria: (1) trials had to deal with NSCLC; (2) investigating the association between serum CYFRA 21-1 level and prognosis; (3) CYFRA 21-1 was clarified as “high” and “low” value; (4) published as a full paper in English for data extraction; and (5) including enough blood sample. To avoid duplication of data, only the most complete and recent study was included. Titles and abstracts of searching records were screened and full text papers were further evaluated to confirm the eligibility. According to the inclusion criteria, two reviewers (Youtao Xu and Mantang Qiu) extracted eligible studies independently, and disagreement between the two reviewers was settled by discussing with the third reviewer (Lei Xu).

### Data extraction

Two reviewers (Xu and Qiu) determined study eligibility independently in order to avoid selection bias in the data abstraction process. The following information was culled from each study: the first author, publication year, country where the research was performed, ethnicity, number of patients, histology, stage, detective method, cutoff, study design, and survival data. The primary endpoint was OS. The studies utilizing PFS were also analyzed.

### Statistical analysis

To evaluate the predictive ability of high serum CYFRA 21-1 level on survival of NSCLC, the hazard ratios (HRs) and their 95% confidence intervals (CIs) of OS and PFS were extracted from eligible studies. If these data were not available, the HRs and CI were calculated according to Tierney' methods^58^. The heterogeneity among studies was evaluated using the Cochran Q and *I*^*2*^ test: for the Q test, a *P* < 0.1 was considered statistically significant^59^. For *I*^*2*^ test, a value >50% was considered a severe heterogeneity^60^, then the random-effects model (based on DerSimonian-Laird method) was used^61^; otherwise, the fixed-effects model (based on Mantel-Haenszel method) was applied. Meta-regression was performed to assess the sources of heterogeneity by ethnicity, surgical intervention, chemotherapy, detective method, cutoff, study design, and sample size (studies with less 200 participants were categorized as “small”, and studies with more than 200 participants were categorized as “large”). Between study, variance Tau-squared (τ2) value was used to evaluate the degree of heterogeneity and describe the extent of heterogeneity explained^62^. Sensitivity analysis was performed to examine the stability of the pooled results. Publication bias was evaluated by a funnel plot with Egger's test and Begg's test, and a *P* < 0.05 was considered significant^63^. All statistical analyses were calculated with STATA software (version 12.0; StataCorp, College Station, Texas USA). And all P values were two-side.

## Supplementary Material

Supplementary Information Supplementary Information

## Figures and Tables

**Table 1:** Characteristics of studies included in this meta-analysis

Author	Year	Country	Ethnicity	Surgery	Chemotherapy	TNM	Design	N	Follow-up(month)	Biomarkers	Method	Cutoff	High/low	HR-E	AHR	ALL	AUL
Lin	2012	China	Asian	Yes	PB/A	IB-IIIA	R	169	53(3–66)	OS	ECLIA	3.30	63/106	HR	1.70	1.19	2.44
Tanaka	2013	Japan	Asian	NA	TKI/M	IIIB/IV	R	160	32.5(23.3–44.6)	PFS/OS	ECLIA	2.00	83/77	HR	1.10	0.85	1.41
Park	2013	Korea	Asian	Yes	NA	I–III	R	298	43.3(0.5–95.6)	PFS/OS	ECLIA	1.95	114/184	HR	1.10	1.01	1.20
Trapé	2012	Spain	Caucasion	No	PB/M	IIIA–IV	P	137	NA	OS	ECLIA	3.30	91/46	HR	2.21	1.40	2.83
Edelman	2012	USA	Mixed	NA	PB/NA	IIIB/IV	P	88	NA	PFS/OS	ECLIA	4.18	NA	SC	1.68	1.33	2.11
Jung	2011	Korea	Asian	NA	TKI/M	IIIB/IV	R	123	NA	PFS/OS	ECLIA	3.30	64/59	HR	2.76	1.38	5.53
Cedrés	2011	Spain	Caucasion	NA	NA	III–IV	R	183	9.7(1–126)	PFS/OS	IRMA	3.30	119/64	SC	1.52	1.06	2.20
Hanagiri	2011	Japan	Asian	Yes	PB/A	I	R	341	42	OS	IRMA	2.00	145/196	HR	2.57	1.21	5.44
Ma	2011	China	Asian	NA	NA	I	R	153	26(2–121)	OS	ECLIA	3.30	41/112	HR	9.81	1.64	58.67
Jin	2010	China	Asian	No	PB/NA	IIIB/IV	R	111	NA	OS	ELISA	3.50	47/64	HR	1.68	1.05	2.69
Takahashi	2010	Japan	Asian	NA	NA	I–IV	R	1202	NA	OS	CLEIA	18.00	NA	HR	2.02	1.14	3.58
Petris	2011	Sweden	Caucasion	NA	NA	I–IV	NA	174	NA	OS	ELISA	6.00	NA	HR	0.80	0.73	1.32
Tomita	2010	Japan	Asian	Yes	NA	I–III	R	291	60.7–141.7	OS	NA	2.40	58/233	HR	1.57	1.29	1.89
Nisman	2009	Israel	Asian	No	NA	IIIA–IV	P	88	NA	OS	ELISA	3.20	60/28	RR	1.80	1.10	2.80
Chen	2010	China	Asian	No	TKI/M	III–IV	R	122	NA	OS	ELISA	3.30	49/73	HR	1.78	1.19	2.65
Chakra	2008	France	Caucasion	NA	NA	I–IV	P	451	NA	OS	IRMA	3.60	224/227	HR	1.50	1.20	1.86
Jacot	2008	France	Caucasion	NA	NA	I–IV	P	289	20.8(2.5–34.1)	OS	IRMA	3.60	92/197	HR	2.30	1.52	3.49
Ishikawa	2008	Japan	Asian	NA	TKI/M	IIIB/IV	R	65	8.3(1.1–37.9)	PFS/OS	ECLIA	2.80	48/17	HR	1.97	0.91	4.27
Nisman	2008	Israel	Asian	No	PB/Mix	IIIA–IV	P	60	NA	OS	ELISA	3.20	43/37	RR	0.50	0.30	0.90
Hisashi	2007	Japan	Asian	Yes	NA	I–IV	R	101	73	OS	CLEIA	3.50	6/95	RR	9.79	2.24	42.80
Matsuoka	2007	Japan	Asian	Yes	NA	I	R	256	35.5(3.7–75.5)	OS	ELISA	2.80	35/221	SC	2.92	2.25	6.84
Mizuguchi	2007	Japan	Asian	Yes	NA	I–IV	R	272	NA	OS	CLEIA	2.00	149/123	HR	2.42	1.99	2.86
Barle'si	2005	France	Caucasion	NA	TKI/NA	IIIB/IV	P	51	NA	OS	ELISA	3.50	13/38	HR	2.45	1.13	5.29
Muley	2004	Germany	Caucasion	Yes	NA	I	R	153	NA	OS	ELISA	3.30	NA	HR	2.16	1.08	4.29
Barlesi	2004	France	Caucasion	No	PB/M	IIIB/IV	P	264	9(1–77)	OS	ELISA	3.50	138/126	HR	1.30	1.06	1.78
Trapé	2003	Spain	Caucasion	No	PB/NA	IIIA–IV	P	48	NA	OS	ECLIA	3.60	NA	HR	1.75	0.93	3.65
Kulpa	2002	Poland	Caucasion	NA	NA	I–IV	NA	200	NA	OS	ECLIA	3.60	127/73	HR	1.31	0.92	1.87
Reinmuth	2002	Germany	Caucasion	Yes	NA	I–IIIA	P	66	42(NA-86)	OS	ELISA	3.57	23/43	SC	2.80	0.98	7.99
Ono	2013	Japan	Asian	No	NA	IIIB/IV	R	284	NA	OS	CLEIA	2.20	134/150	HR	0.43	0.31	0.59
Ondrej F	2014	Czech	Caucasion	NA	TKI/M	IIIB/IV	R	144	NA	OS/PFS	ELISA	2.50	83/61	HR	2.17	1.48	3.19
Monik	2014	NA	Gaussian	Yes	None	I–IV	P	50	72.5(4–108)	OS	ELISA	2.10	0.45	SC	1.00	0.45	2.21

HR-E: HR Estimate; R: retrospective; P: prospective; NA: not available; PB/A: Platinum-based/adjuvant chemotherapy; PB/M: Platinum-based/metastatic chemotherapy; PB/mix: Platinum-based/adjuvant chemotherapy and metastatic chemotherapy; TKI/M: TKI/metastatic chemotherapy; TKI/Mix: TKI/adjuvant and metastatic chemotherapy; SC: survival curve; AHR: adjusted hazard ratio; ALL: adjusted lower limit; AUL: adjusted upper limit; ECLIA: electrochemiluminescence immunoassay; ELISA: enzyme-linked immunosorbent assay; CLEIA: chemiluminescent enzyme immunoassay; IRMA: immunoradiometric assay.

**Table 2:** Main results of the meta-analysis

	N.of studies	N. of patients	HR(95%CIs)		Heterogeneity test	
				Q	I-squared	P-value
OS						
Overall	31	6394	1.60(1.36,1.89)	204.53	85.30%	<0.001
Stage						
I–IIIA	6	1138	2.18(1.70,2.80)	5.99	16.60%	0.307
IIIB–IV	9	1290	1.47(1.02,2.11)	68.04	88.20%	<0.001
Mix(I–IV)	16	3966	1.52(1.24,1.88)	116.06	87.10%	<0.001
Surgical intervention						
Surgery	10	1997	1.94(1.42,2.67)	85.90	89.50%	<0.001
Non-surgery	8	1114	1.24(0.79,1.95)	70.54	90.10%	<0.001
Chemotherapy						
EGFR-TKI	6	665	1.83(1.31,2.58)	14.84	66.30%	0.011
Platinum-based	8	1218	1.53(1.18,1.99)	24.67	71.60%	0.001
Ethnicity						
Asian	17	4096	1.62(1.25,2.09)	160.26	90.00%	<0.001
Caucasian	13	2210	1.60(1.31,1.95)	36.48	67.10%	<0.001
Sample size						
Small	20	2246	1.66(1.35,2.03)	68.62	72.30%	<0.001
Large	11	4148	1.53(1.16,2.00)	130.45	92.30%	<0.001
Study design						
Prospective	11	1542	1.58(1.27,1.96)	30.18	66.90%	0.001
Retrospective	18	4478	1.75(1.38,2.22)	155.27	89.10%	<0.001
PFS						
Overall	7	1061	1.41(1.19,1.69)	24.54	75.60%	<0.001

OS: overall survival; HR: hazard ratio; CI: confidence interval; PFS: progression-free survival.
