# Supplementary material for: Prognostic value of serum cytokeratin 19 fragments (Cyfra 21-1) in patients with non-small cell lung cancer
Source: Sci Rep. 2015 Apr 22;5:9444. doi: 10.1038/srep09444 (PMC5386115; doi:10.1038/srep09444)
Supplement: Supplementary Information [file srep09444-s1.pdf]

Prognostic value of serum cytokeratin 19 fragments (Cyfra 21-1) in patients with non-small cell lung cancer

Youtao Xu[1,2#]; Lei Xu[3#]; Mantang Qiu[1,4#]; Jie Wang[1]; Qing Zhou[1,5]; Lin Xu[1]; Jian Wang[3]; Rong Yin[1].

Institution:

1 Department of Thoracic Surgery, Nanjing Medical University Affiliated Cancer Hospital, Jiangsu Key Laboratory of Molecular and Translational Cancer Research, Cancer Institute of Jiangsu Province, Baiziting 42, Nanjing, P.R. China, 210009

2 The First Clinical College of Nanjing Medical University, Nanjing, 210000, China

3 Department of Thoracic Surgery, The Affiliated Jiangning Hospital of Nanjing Medical University, No. 168 Dongshan Street Gushan Road, Nanjing 211100, China

4 The Fourth Clinical College of Nanjing Medical University, Nanjing 210000, China

5 Department of Oncology, Nanjing Medical University Affiliated Cancer Hospital, Baiziting 42, Nanjing, P.R. China, 210009

#the three authors contributed equally to this work

Correspondence: Lin Xu

Address: Baiziting 42, Nanjing, China, 210009

TEL: 86-25-83284700

FAX: 86-25-83641062

E-mail: xulin83cn@gmail.com

This work can also correspondence to Rong Yin; TEL:86-25-83283408, E-mail: yinhero001@126.com; Jian Wang; TEL:86-25-52281471, E-mail: wangjianjnyy@126.com.

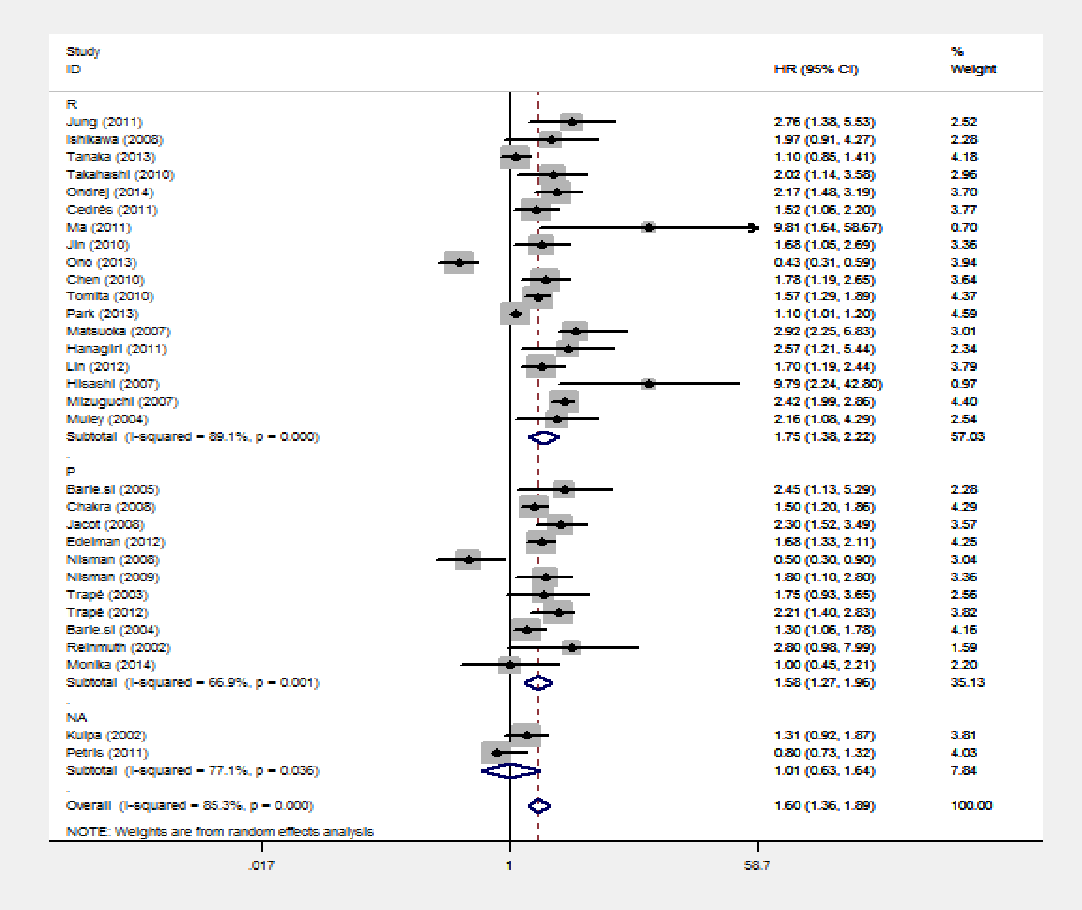

Supplementary Fig. S1 The association between serum CYFRA 21-1 and overall survival of NSCLC stratified by study design

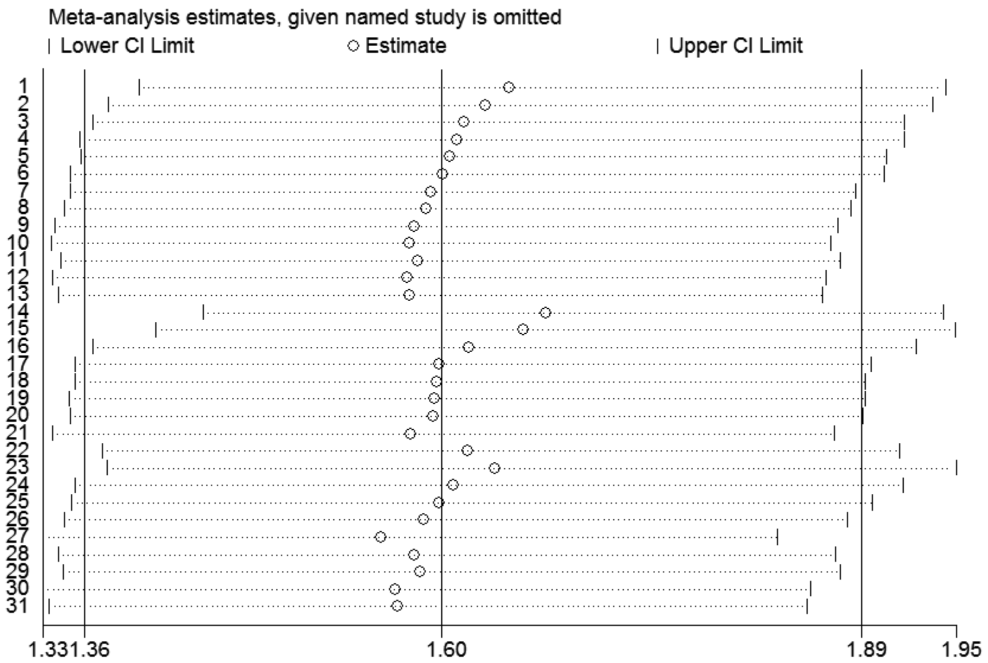

Supplementary Fig. S2 Sensitivity analysis of association between serum CYFRA 21-1 and overall survival of NSCLC

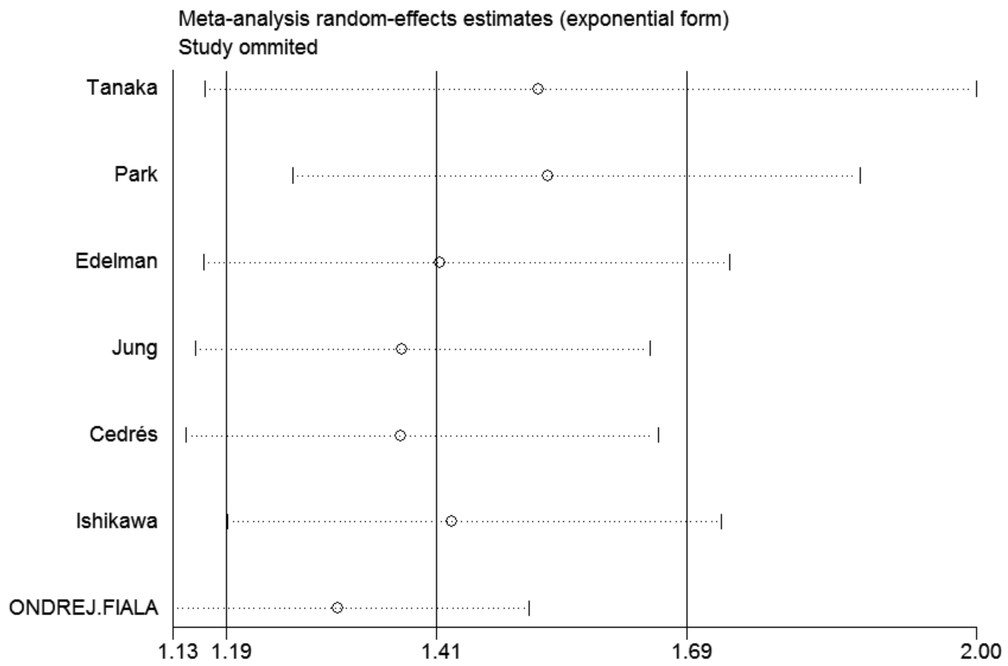

Supplementary Fig. S3 Sensitivity analysis of association between serum CYFRA 21-1 and progression free survival of NSCLC
